# *In vivo* effect of mouthwashes on viable viral load of SARS-CoV-2 in saliva: a pilot study

**DOI:** 10.1080/20002297.2023.2198432

**Published:** 2023-04-11

**Authors:** Alvaro Sánchez Barrueco, María Victoria Mateos-Moreno, José Miguel Villacampa Aubá, Alfonso Campos González, Abel Bogoya Castaño, Raúl Rubio Yanguas, Asier Blanco Goñi, Javier Zapardiel Ferrero, Carlos Cenjor Español, Verónica Ausina Márquez, Sandra García-Esteban, Alejandro Artacho, F. Xavier López Labrador, Alex Mira, María D. Ferrer

**Affiliations:** a ENT and Cervicofacial Surgery Department, Fundación Jiménez Díaz University Hospital, Madrid, Spain; b ENT and Cervicofacial Surgery Department, Villalba General University Hospital, Collado Villalba, Spain; c Department of Dental Clinical Specialties, School of Dentistry, Madrid Complutense University, Madrid, Spain; d Microbiology Department, Fundación Jiménez Díaz University Hospital, Madrid, Spain; e Microbiology Department, Villalba General University Hospital, Collado Villalba, Spain; f Department of Dentistry, European University of Valencia, Valencia, Spain; g Genomics & Health Department, FISABIO-Public Health Foundation, Valencia, Spain; h Department of Microbiology and Ecology, Medical School, University of Valencia, Valencia, Spain; i CIBER in Epidemiology and Public Health (CIBERESP), Instituto de Salud Carlos III, Madrid, Spain

**Keywords:** SARS-CoV-2, saliva, COVID-19, infectivity, mouthwash, chlorhexidine, cymenol

## Abstract

Current data on the efficacy of antiseptic mouthwashes to reduce viral load are contradictory. Firstly, *in vitro* data indicate very strong virucidal effects that are not replicated in clinical studies. Secondly, most clinical studies identify a limited effect, do not include a control/placebo group, or do not evaluate viral viability in an infection model. In the current manuscript, we perform a double-blind, randomized clinical trial where salivary viral load was measured before and after the mouthwash, and where saliva samples were also cultured in an *in vitro* infection model of SARS-CoV-2 to evaluate the effect of mouthwashes on viral viability. Our data show a 90–99% reduction in SARS-CoV-2 salivary copies with one of the tested mouthwashes, although we show that the remaining viruses are mostly viable. In addition, our data suggest that the active ingredient concentration and the overall excipients’ formulation can play an important role; and most importantly, they indicate that the effect is not immediate, being significant at 15 min and having maximum effectiveness after 1 h. Thus, we show that some oral mouthwashes can be useful in reducing viral transmission, although their efficacy must be improved through refined formulations or revised protocols.

## Introduction

Although the current epidemiological data are less alarming in terms of both trends of associated death and hospitalization admission, almost 3 years after the start of the COVID-19 pandemic, its impact is still significant worldwide, reaching more than 634 million new cases diagnosed and over 6,6 million deaths [1]. Given these figures, research focused on the development of new measures to help contain the spread of the pathogen and prevent its transmission is more than justified. Although the most widespread diagnosis is through the antigen test of nasal samples or RT-qPCR of nasopharyngeal samples, it has been shown that saliva is a valid sample for detection and has the advantage of being less invasive. When doing qPCR with the same primers, saliva and nasopharyngeal Ct values are significantly correlated [2], and several studies have even shown that saliva is more sensitive for SARS-CoV-2 detection than nasopharyngeal swabs, especially at the beginning of the infection [3].

In this context, salivary droplets may represent the main source of the human-to-human transmission of SARS-CoV-2 infection [4]. Therefore, the prevention of the formation of these infectious saliva droplets would greatly facilitate the control of the epidemic by slowing down the spread of the virus.

On this basis, the use of antiseptic mouthwashes as a measure to prevent infection against SARS-CoV-2 has been evaluated in numerous *in vitro* studies [5–9]. In fact, its use has been suggested in dental clinics and in maxillofacial operations prior to the procedure by the specialist, even though the results of the different studies to date have not shown congruent results regarding its effectiveness.

On the other hand, almost all clinical trials carried out to determine the efficacy of oral mouthwashes have quantified the effect on the reduction of viral load in saliva by RT-qPCR, which would be a measure of viable and non-viable viruses, but without specifically considering the outcome of reduced infectivity or virucidal capacity. In this regard, the use of Vero-E6 cell lines has already been demonstrated as a useful method to measure viral load, as well as their infective capacity [2]. However, there is a wide patient- and sample-dependent variability that must be evaluated to obtain conclusive results.

In a previous study from our team [2] the effect of four oral rinses was analyzed at 30 min and 60 min after rinsing, both in terms of total viral load and, for the first time, in terms of infectivity in cell cultures. In the present study, we intend to evaluate the immediate effect of the mouthwash, analyzing saliva samples at 5 and 15 min, as well as 1 hour after rinsing with chlorhexidine at two different concentrations (0.12% and 0.2%), and a newly studied mouthwash containing Cymenol ZnCl_2_. Thus, the goal of the current work is to determine if the effect of the rinse is immediate, in addition to the effective time window to suggest safe treatment guidelines directed towards mitigating the spread of the disease.

## Methods and materials

### Study rationale and design

The *in vivo* effect of several antiseptics on the viability of SARS-CoV-2 in saliva samples of COVID-19 patients was evaluated based on a randomized, double-blind, four-parallel-group, placebo-controlled, multicentre trial (ClinicalTrials.gov Identifier: NCT04707742). The study was performed in Fundación Jiménez Díaz University Hospital (Madrid, Spain) and Villalba General University Hospital, after approval by Fundación Jiménez Díaz University Hospital Ethical Committee on 2021/04/13 with code ER3_EO095-20_FJD-HGV-HIE.

Participants were adults hospitalized for different diagnoses. Each patient signed a written consent and met the inclusion criteria: a SARS-CoV-2 RT-PCR nasopharyngeal swab sample positivity and the capability to complete a mouthwash rinse and donate 2 mL of saliva for each sample. Exclusion criteria include the use of an antiseptic mouthwash for 2 days before the start of the study and synchronous participation in a COVID-19 study testing experimental drugs, as well as any allergy or hypersensitivity to the mouthwash components.

### Eligibility and data collection

In order to increase the detectability of a potential improvement and to guarantee subsequent cell culture [2], only patients with nasopharyngeal samples showing Ct values of the RT-qPCR SARS-CoV-2 E-gene≤30 were selected. Data such as age and gender of patients, time of symptoms’ onset and previous SARS-COV-2 vaccination information were collected.

### Randomisation and masking

Each participant was serially assigned to a code consisting of a patient number and a letter for each of the four treatment groups (A, B, C and D) from a randomised table, unknown to the research team who performed RNA extraction, RT-qPCR and data analysis. There were no differences in the type of tubes, labelling and volume of mouth rinses and placebo to achieve masking.

### Procedures

The four different mouthwashes studied were randomized into four treatment groups: Group A (chlorhexidine [CHX] 0.12%), Group B (CHX 0.2%), Group C (Cymenol + ZnCl_2_ [Cym ZnCl_2_]) and Group D (placebo, distilled water). The concentrations of mouthwashes A, B and C were those commercially established by the manufacturer (Lacer Chlorhexidine Mouthwash© 0.12%, Lacer Chlorhexidine Mouthwash© 0.20%, Gingilacer Delicate Gums Mouthwash©). Distilled water was selected as a placebo based on previous studies reporting that distilled water had no virucidal effect on SARS-CoV-2 [10].

**Figure 1.**
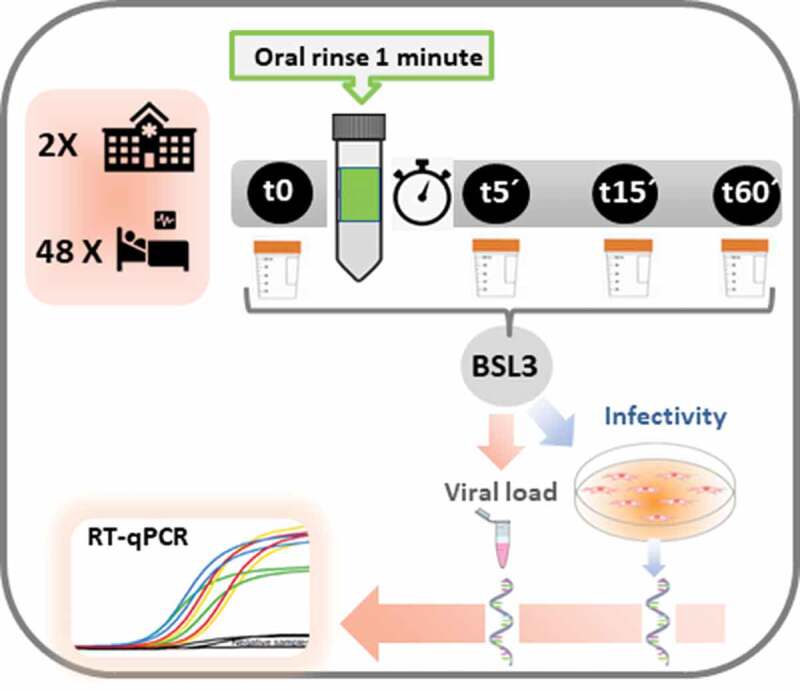
Clinical trial protocol. From top to bottom, the upper left box represents the number of hospitals participating in the study and the number of patients randomised. The timeline of non-stimulated saliva samples collection is shown to the right, including the baseline sample before mouth rinse (t0), and 5 (t5´), 15 (t15´) and 60 minutes (t60´) samples after the one-minute oral rinse. In the Biosafety Level 3 laboratory (BSL3), all samples were divided into two aliquots, one to determine the viral load per mL of saliva (by RNA extraction and subsequent RT-qPCR), and the other to assess viral infectivity in the Vero-E6 cell culture and subsequent viral replication, quantified by RNA extraction and RT-qPCR of the viral culture supernatant.

Each participant was asked to donate four saliva samples of at least 2 mL: one at baseline before rinsing, and the other three at 5, 15 and 60 min after mouthwash (Figure 1). Each sample provided by the patient was collected using the drooling technique in a sterile millimetre plastic tube.

Before the procedure, participants were reminded that they could not drink anything other than water, in addition to not eating or brushing their teeth for 1 hour before sample collection and until the end of the sampling. After collecting the baseline saliva sample (t0), each patient rinsed with the randomised mouthwash (15 mL) for 1 min. At 5 min (t5), 15 min (t15) and 60 min (t60) after rinsing, respectively, at least 2 mL of saliva were collected per sample. Then, the plastic tube was labelled with the code assigned to the patient and the time point, and kept hermetically sealed at −80ºC. All samples were transferred to the Biosafety Level 3 (BSL-3) laboratory on dry ice according to UN3733 standards.

### Sample processing

Samples were thawed at room temperature, and a 200 uL aliquot of each was used for RNA extraction. The rest of each sample was stored at −80C until further testing in Vero-E6 cell lines. RNA extraction was performed following Sánchez Barrueco *et al.* [2]. To detect the SARS-CoV-2 E gene, a multiplex RT-qPCR test was performed based on WHO-Charité and U.S. CDC assays [11,12], following the details of the protocol described by Mira-Iglesias *et al.* [13].

### SARS-CoV-2 culture from saliva samples in Vero-E6 cells

Saliva samples were cultured following the details of the protocol described by Sánchez Barrueco *et al.* [2]. Briefly, saliva samples diluted 1:1 in PBS were centrifuged, and 300 uL of the supernatant were incubated with Vero-E6 (ATCC) cells for 1 hour to allow absorption of live viruses. Then, saliva (with unabsorbed viruses) was removed and replaced by infection media and incubated for 5 days. After 5 days of infection, both supernatants were collected for RNA extraction, and cytopathic effect (CPE) was recorded as negative or positive [14].

### Statistical analysis

A Wilcoxon test was used to assess mean differences between study time points and between different mouthwashes or study arms. Spearman’s correlation analyses were performed to assess the relationship between clinical variables with baseline salivary viral load, viable load, and nasopharyngeal viral load. All calculations and tests were performed with R software (version 3.6.3, ‘stats’ package) [14].

## Results

After ethical approval, patients began to be recruited from March 13^th^, 2021. The low incidence of the disease in Spain during the summer and autumn months caused a delay in the estimated recruitment time. However, the increase in incidence at the end of the year allowed the recruitment of the rest of the patients required for the study (*n* = 48). Recruitment was completed on January 8^th^, 2022.

**Figure 2.**
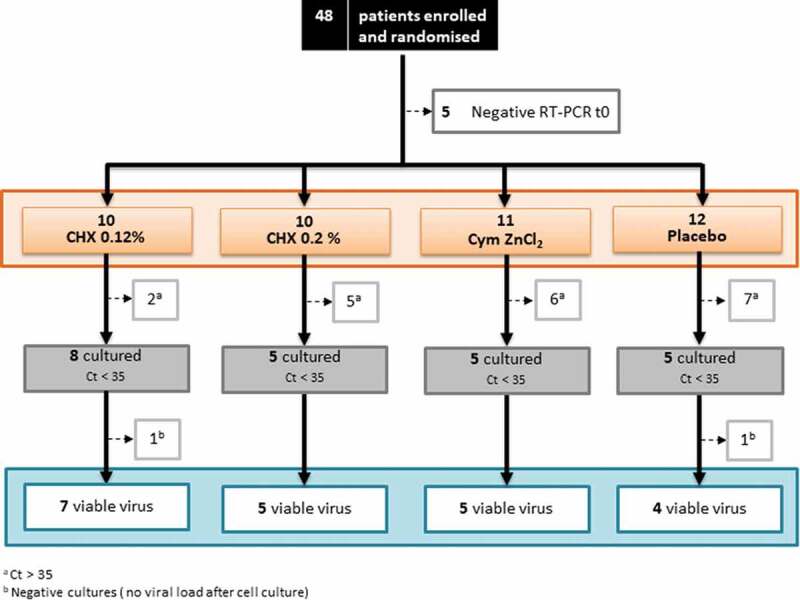
Flow diagram of the patients included in the study. From top to bottom, the flow chart illustrates the number of patients enrolled and randomized, excluded for negative RT-qPCR in the baseline samples and inpatients assigned to each of the different treatment groups (CHX 0.12%, CHX 0.2%, Cym ZnCl_2_ and Placebo) in which total viral load (orange square) and viable (infective) viral load (gray square) were assessed. Blue squares indicate the number of patients for each treatment group in whom viruses were successfully amplified in cell culture. a (Number of patients excluded from the cell culture assays for low salivary viral load [Ct value>35 in the RT-qPcrs in the baseline saliva sample]). b (Number of patients without detectable viral load after cell culture in the baseline saliva sample).

Of the 48 patients enrolled and randomized Figure 2, 43 were analyzed at all time-points (t0-basal sample, t5, t15 and t60 minutes after rinsing, respectively) for their saliva viral load by RNA extraction and RT-qPCR. The remaining five patients were excluded from the analysis because viral load in saliva sample prior to rinsing (t0) was not detected. The number of patients distributed by the treatment group for which the viral load was analyzed is highlighted in orange in Figure 2.

In those patients whose baseline Ct values in saliva viral load (t0) were <35, viral viability was further evaluated by cell culture assays, based on previous results about successful isolation of virus in cell culture [15]. Thus, the virus load viability from 92 saliva samples corresponding to a total of 23 individuals at all evaluated time points was tested using cell culture assays, with a sample size of at least 5 patients per group (gray box in Figure 2). Following the exclusion criterion of the maximum Ct threshold value obtained in the viral load, more than 90% of the cultured samples were positive (21 of the 23 patients evaluated), being able to isolate SARS-CoV-2 from a sample size of 4–7 individuals per group of treatment (blue box in Figure 2).

**Figure 3.**
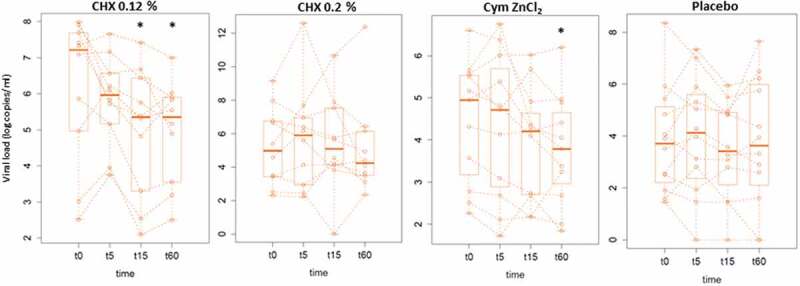
Salivary viral load. Median values of viral load expressed in log copies per mL of saliva measured by RT-qPCR for baseline sample (t0), 5 min (t5), 15 min (t15), and 60 min (t60) after mouthwash, respectively, are displayed for each treatment group: CHX 0.12%, CHX 0.2%, Cym ZnCl_2_, and Placebo. Values from the same patient are linked by dotted lines. For clarity, different scales of the y-axis are used.

The box plots in Figure 3 show the results obtained for total viral load (live and dead viruses) in saliva for each of the four treatment groups at the different time-points tested, namely basal saliva (t0), 5 min (t5), 15 min (t15) and 60 min (t60) after mouthwash, respectively.

* Significant Wilcoxon’s paired test with respect to baseline saliva (t0) (p-value <0.05).

Both in the CHX 0.12% group and in the Cym ZnCl_2_ group, a progressive decrease in salivary viral load was observed over time, being significant with respect to the baseline from 15 min after the mouthwash in the CHX 0.12% group (p-value = 0.037) and with a trend towards significance in cymenol (p-value = 0.054). In addition, the values were significantly different at 1 hour after the mouthwash in both groups (CHX 0.12% group: p-value = 0.02; Cym ZnCl_2_ group: *p*- value = 0.04). In contrast, in both the placebo group and the CHX 0.2% group, no differences were observed between time-points.

Treatment with CHX 0.12% produced a decrease of 1.2 log units in mean salivary viral load values at 15 min after rinsing and was maintained up to 1 h, which is equivalent to a significant 94% decrease in total salivary load from 15 min after rinsing. In the case of Cym ZnCl_2_, the average decrease was 0.7 log units, so the viral load was reduced by almost 80% at 1 hour after rinsing.

**Figure 4.**
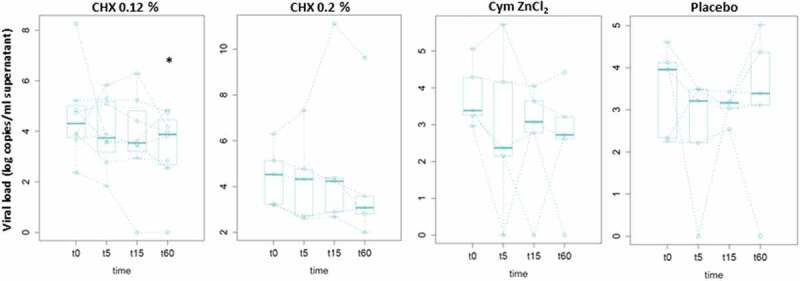
Infective viral load. Median values of SARS-CoV-2 genome copies in cell culture supernatants log (copies/mL) after 5 days of saliva culture in Vero-E6 cells measured by RT-qPCR are represented in the box plots for each treatment group (CHX 0.12%, CHX 0.2%, Cym ZnCl_2_, and Placebo) at different time points (t0 for basal and t5, t15 and t60 for 5, 15 and 60 min after the mouthwash, respectively). The dotted lines join the values of the same patient over time. For clarity, different scales of the y-axis are used.

To determine the viral viability in the saliva samples, infectivity assays were performed using saliva culture in Vero-E6 cells for 5 days. The results of SARS-CoV-2 genome copies/mL in the cell supernatant at day 5 for each of the study groups at every time-point are shown in Figure 4.

* Significant Wilcoxon’s paired test with respect to the baseline saliva (t0) (p-value <0.05).

In contrast with the viral load results, the median of viable virus decreased in all treatment groups after rinsing, including the placebo group.

On the other hand, although the largest decrease in mean values of viable virus was observed in the Cym ZnCl_2_ group at 15 min and 1 hour after mouthwash (relative decrease to mean baseline levels of 1.7 and 1.9 log genome copies/mL of culture supernatant, respectively), the values were not statistically significant. Instead, a significant decrease in mean infective viruses **was only observed** in the CHX 0.12% group at 1 hour after rinsing (p-value = 0.04) compared to baseline values. **This significant decrease was 1.2 log genome copies/mL of culture supernatant**, which is equivalent to a 94% reduction in infectivity 1 hour after the oral rinse **with 0.12% CHX**. In contrast, no significant differences in virus infectivity were found for the other treatments.

In conclusion, Cym ZnCl_2_ reduced the total salivary viral load of SARS-CoV-2 by 80% 1 hour after rinsing, but despite the fact that our results show a positive correlation between the number of viable viruses recovered and the total salivary load (Spearman’s correlation coefficient = 0.4; p-value = 9.3e-5), no significant effect was observed on the number of viable viruses recovered. On the other hand, CXH 0.12% affected the total viral load from 15 min after the mouthwash and was maintained until 1 hour, when it also produced a 94% infectivity reduction in our *in vitro* infection system.

The information on the clinical variables collected from each patient in each treatment group is summarized in Table 1. The percentage of men in each treatment group was higher than that of women (67% men vs. 33% women), except in the Cym ZnCl_2_ group. The mean age of the patients per group was between 56 and 65 years. The mean number of days since the last nasopharyngeal PCR was performed at the hospital and the number of days of symptoms for each study group was between 2–3 days and 5–13 days, respectively. Finally, the number of patients with at least one dose of SARS-CoV2 vaccine was between 5 and 9.

**Table 1.** Clinical characteristics of the patients.

		CHX 0.12% (*n* = 12)	CHX 0.2% (*n* = 12)	Cym ZnCl_2_ (*n* = 12)	Placebo (*n* = 12)
Sex	Female	33%	33%	67%	33%
	Male	67%	67%	33%	67%
Age (years)		65 (85–30)	59 (85–36)	56 (87–24)	58 (86–26)
Days from last PCR		3 (13–0)	2 (0–3)	2 (1–4)	2 (1–3)
Symptom days		5 (2–7)	13 (90–2)	6 (2–10)	7 (0–11)
Vaccinated		9	9	7	5

There was no statistically significant correlation between any of the clinical variables with baseline salivary viral load, viable load, or nasopharyngeal viral load.

## Discussion

Different protocols have been established in dental clinics as a preventive measure to avoid the dispersion and contagion of SARS-CoV-2, such as rinsing with oral mouthwashes as a prior step to any clinical inspection [16,17]. However, the studies on which these decisions have been based show contradictory evidence and certain limitations. Firstly, conditions tested in *in vitro* studies do not mimic natural conditions, and caution should be exercised in extrapolating these results to in vivo models. For instance, they do not take into account the rinsing shearing effect, the outcome of saliva clearance or the influence of different salivary compounds, among others. Therefore, *in vitro* studies are a necessary approach to identify potential compounds of interest, but it is essential that rigorous in vivo clinical trials are performed in order to establish medical or social protocols for action. In this sense, unfortunately, many of the in vivo studies show disparate or non-repetitive results between them [2,18–20]. In addition, a relevant feature of the vast majority of in vivo studies published to date is that they have been based exclusively on the total viral load measured by RT-qPCR [12,18,21–24]. Although this quantification method is highly standardized, a great disadvantage is that it does not allow evaluating the effect of mouthwashes on the population of infective or viable viruses. This means, for example, that a negative result in total viral load reduction does not imply a lack of mouthwash efficacy. Therefore, claims based solely on total viral load data should be considered with caution.

There are currently three clinical trials in which the effect on viral load infectivity has also been evaluated besides total viral load [2,19,20]. On the one hand, Alemany *et al*. used ELISA-based methodology to quantify the viral nucleocapsid to detect the disruption of the viral envelope as a proportion of lysed virus in relation to the basal levels [20]. In two other studies, the viability of the viral load was determined directly by cell culture infection of the saliva samples and subsequent RT-qPCR after several days post-infection [2,19]. In both approaches, viability quantification in cell culture and, to a greater extent, viability loss quantification by ELISA, are indirect measures of viral load infectivity. Nevertheless, they undoubtedly continue to provide a more reliable approximation for the evaluation of viral infectivity than the total viral load measured exclusively by RT-qPCR.

The lack of *in vivo* studies in which the effectiveness of mouthwashes is evaluated based on viral infectivity largely derives from the difficulty of obtaining positive viral cultures from saliva samples [25,26]. For example, in the study by Meister *et al*. in which the effect of benzalkonium chloride was evaluated, positive cultures were only obtained in six out of the 32 total patients [19]. In our case, the high proportion of positive cultures obtained (up to 100% in the samples of the CHX 0.12% and Cym ZnCl_2_) is probably due to the restrictions in sample selection, especially the CT values. Previous results from our group [2] showed that the viral culture was negative in almost 80% of the samples with SARS-Co-V RNA RT-PCR Ct values higher than 31. Therefore, despite an added effort for patient recruitment, the infection protocol in Vero-E6 cells set up here appears to be a feasible method to assess viral infectivity. Nevertheless, in up to more than half of the patients in one of the groups, no positive basal viral load cultures were obtained, considerably limiting the sample size in our study. Thus, further improvements and more appropriate cell lines for SARS-CoV-2 infection could probably increase viral culture sensitivity in the future. A larger population size will also be needed to confirm our results, as considerable inter-individual variability in mouthwash efficacy has been detected [18,25,26], limiting data interpretation in clinical studies with small sample size.

In the current work we have focused on determining the mouthwash effect on the viable and total viral load in short times after rinsing, simulating the safety protocol in clinical settings. The results show that a minimum action time greater than 15 min is necessary to observe a significant decrease in the viable viral load. Therefore, it should be emphasized that, at least with the mouthwashes tested, it would be necessary to modify the action protocols and extend the waiting time between rinsing and oral inspection by the professional. In addition, the results disclose that a higher concentration of the compound does not imply a greater effect. In fact, significant results of decreased infective viral load were only observed in the group treated with CHX 0.12% and not in the group with CHX 0.2%. This suggests that more than the active ingredient, it is necessary to evaluate the product together with all its components, since these can show an effect *per se*, or generate a synergistic effect with the active compound or with the rest of the ingredients. For example, the effect of a different CHX formulation was evaluated in previous studies, but no significant differences were observed [2,18]. The significant effect obtained with another commercially available Chlorhexidine mouthwash at 0.12%, in the current study, highlights the need to test not only the active ingredient but rather the overall effect of its combination with the excipients included in the final formulation. For example, xylitol is a common excipient in oral mouthwashes because of its known effect against tooth decay, but recent work demonstrates that this compound has virucidal activity against several viruses, including SARS and influenza [27]. The different effect obtained by chlorhexidine at two different concentrations also underlines the importance of an appropriate dose.

On the other hand, the *in vivo* effect of Cym ZnCl_2_ has been evaluated for the first time. Our results showed a significant reduction of total viral load 1 hour after rinsing, although no significant effect was observed on the viability of the remaining viruses. This suggests that within the tested formulation, cymenol may not be a good candidate for an immediate effect in the prevention of SARS-CoV2 infectivity.

In relation to other compounds in which their effectiveness has been evaluated *in vivo*, it is worth mentioning Cetylpyridinium chloride (CPC). Both in our previous work [2] and in that of Alemany and collaborators [20], a significant effect on the reduction of infectivity was detected 1 hour after rinsing, but not at 30 min. Future studies should therefore evaluate the combination of this and other compounds on an immediate effect in the prevention of SARS-CoV2 infectivity, which would largely expand its practical application in clinical settings [28].

In conclusion, the results from our pilot study show that the effect of the tested mouthwashes on total viral load is moderate, decreasing the salivary viable viral load by 94% with the Chlorhexidine 0.12%, which corresponding to less than 2 logs decrease. Interestingly, the remaining viral particles also showed a significant decrease in viability, but only 1 hour after rinsing, and our work did not test the effect of repeated mouthwashes. In this sense, studies focused on determining the possible cumulative effect after several rinses or the combination of different consecutive treatments could shed light on the development of effective and safe action protocols. Until then, we recommend maintaining strict preventive measures and we hope that the current study stimulates further work along these lines.
